# Supplementary material for: Heat adaptation of phage T7 under an extended genetic code
Source: Virus Evol. 2021 Dec 1;7(2):veab100. doi: 10.1093/ve/veab100 (PMC8923235; doi:10.1093/ve/veab100)
Supplement: veab100_Supp [file veab100_supp.zip › Sequencing_Analysis.zip!/Sequencing_Analysis/S2/evidence/RA_27.html]

BRESEQ :: Evidence


| Read alignment evidence... | | | | | | | | | | | |
| --- | --- | --- | --- | --- | --- | --- | --- | --- | --- | --- | --- |
|  | seq id | position | | ref | new | freq | score (cons/poly) | reads | annotation | genes | product |
| \* | NC\_001604.1 | 33,023 | 0 | A | G | 25.0% | 9424.4 / inf | 6553 | intergenic (–/–) | *–/–* | –/– |
| Reads supporting (aligned to +/- strand):  **ref** base A (2533/2334);  **new** base G (864/758);  **total** (3441/3112) | | | | | | | | | | | |
| Fisher's exact test for biased strand distribution *p*-value = 4.05e-01 | | | | | | | | | | | |
| Kolmogorov-Smirnov test that lower quality scores support variant *p*-value = 9.97e-01 | | | | | | | | | | | |

Only 100 of 6560 total aligned reads displayed.  

`TGAGATGCTTAAGGAATTACACGGGGTGAAGGAAGTAACACCAGAGATGGTAGAGAAGTACGCTATGGATAAGGCTTATGGTATCTCCCACTCAGACCAGTTCACCAACAGTTCCATAATAGAAGAGAACATTGAGGGCTTAGTAGGTATCGAGAATAACTCATTCCTTGAGGCACGTAACTTGTTTGATTCGGACCTATCCATCACTATGCCAGACGGACAGCAATTCTCAGTGAATGACCTAAGGGACTTCGATATGTTCCGCATCATGCCAGCGTATGACCGCCGTGTCAATGGTGACATCGCCATCATGGGGTCTACTGGTAAAACCACTAAGGAACTTAAGGATGAGATTTTGGCTCTCAAAGCGAAAGCTGAGGGAGACGGTAAGAAGACTGGCGAGGTACATGCTTTAATGGATACCGTTAAGATTCTTACTGGTCGTGCTAGACGCAATCAGGACACTGTGTGGGAAACCTCACTGCGTGCCATCAATGACCTAGGGTTCTTCGCTAAGAACGCCTACATGGGTGCTCAGAACATTACGGAGATTGCTGGGATGATTGTCACTGGTAACGTTCGTGCTCTAGGGCATGGTAT  >  NC_001604.1/32724‑33323`  

                                                                                                                                                                                                                                                                                                           `|`                                                                                                                                                                                                                                                                                                              
                  `acacGGGGTGAAGGAAGTAACACCAGAGATGGTAGAGAAGTACGCTATGGATAAGGCTTATGGTATCTCCCACTCAGACCAGTTCACCAACAGTTCCATAATAGAAGAGAACATTGAGGGCTTAGTAGGTATCGAGAATAACTCATTCCTTGAGGCACGTAACTTGTTTGATTCGGACCTATCCATCACTATGCCAGACGGACAGCAATTCTCAGTGAATGACCTAAGGGACTTCGATATGTTCCGCATCATGCCAGCGTATGACCGACGTGTCAATGGTGGCATAGCAATCATGGGGACt                                                                                                                                                                                                                                                                                           >  1:392655/1‑301 (MQ=255)`  
                       `agatgtggataagagacagagagATGGGAGAGAAGTACGCTATGGATAAGGCTTATGGTATCTCCCACTCAGACCAGTTCACCAACAGTTCCATAATAGAAGAGAACATTGAGGGCTTAGTAGGTATCGAGAATAACTCATTCCTTGAGGCACGTAACTTGTTTGATTCGGACCTATCCATCACTATGCCAGACGGACAGCAATTCTCAGTGAATGACCTAAGGGACTTCGATATGTTCCGCATCATGCCAGCGTATGACCGCCGTGTCAATGGTGACATCGCCATCATGGGGTCTACTgg                                                                                                                                                                                                                                                                                      <  1:462166/282‑1 (MQ=255)`  
                                `agagacagcgagagATGGTAGAGAAGTACGCTATGGATAAGGCTTATGGTATCTCCCACTCAGACCAGTTCACCAACAGTTCCATAATAGAAGAGAACATTGAGGGCTTAGTAGGTATCGAGAATAACTCATTCCTTGAGGCACGTAACTTGTTTGATTCGGACCTATCCATCACTATGCCAGACGGACAGCAATTCTCAGTGAATGACCTAAGGGACTTCGATATGTTCCGCATCATGCCAGCGTATGACCGCCGTGTCAATGGTGGCATCGCCATCATGGGGTCTACTGGTAAAACCAc                                                                                                                                                                                                                                                                             <  1:763286/291‑1 (MQ=255)`  
                                   `tAACACCAGAGATGGTAGAGAAGTACGCTATGGATAAGGCTTATGGTATCTCCCACTCAGACCAGTTCACCAACAGTTCCATAATAGAAGAGAACATTGAGGGCTTAGTAGGTATCGAGAATAACTCATTCCTTGAGGCACGTAACTTGTTTGATTCGGACCTATCCATCACTATGCCAGACGGACAGCAATTCTCAGTGAATGACCTAAGGGACTTCGATATGTTCCGCATCATGCCAGCGTATGACCGCCGTGTCAATGGTGACATCGCCATCATGGGGTCTACTGGTAAAAccctgtc                                                                                                                                                                                                                                                                          >  1:276102/1‑296 (MQ=255)`  
                                     `acacCAGAGATGGTAGAGAAGTACGCTATGGATAAGGCTTATGGTATCTCCCACTCAGACCAGTTCACCAACAGTTCCATAATAGAAGAGCACATTGAGGGCTTAGTAGGTATCGAGAATAACTCATTCCTTGAGGCACGTAACTTGTTTGATTCGGACCTATCCATCACTATGCCAGACGGACAGCAATTCTCAGTGAATGACCTAAGGGACTTCGATATGTTCCGCATCATGCCAGCGTATGACCGCCGTGTCAATGGTGACATCGCCATCATGGGGTCTACTGGTAAAACCACTAAgg                                                                                                                                                                                                                                                                        <  1:320503/301‑1 (MQ=255)`  
                                     `acacCAGAGATGGTAGAGAAGTACGCTATGGATAAGGCTGATGGTATCTCCCACTCAGACCAGTTCACCAAAAGTTACATAATAGAAGAGAACATTGAGGGCTTAGTAGGTATCGAGAATAACTCATTCCATGAGGCACGTAACTTGTTTGATTCGGACCTATCCATAACTATGCCAGACGGACAGAAATTCTCAGTGAATGACCTAAGGGACTTCGATATGTTCCGCATCATGCCAGCGTATGACCGCCGTGTCAATGGTAACATCGACATCATGGGGTCTACTGGTAAAACCACTAAgg                                                                                                                                                                                                                                                                        <  1:440220/301‑1 (MQ=255)`  
                                             `gaTGGTAGAGAAGTACGCTATGGATAAGGCTTATGGTATCTCCCACTCAGACCAGTTCACCAACAGTTACATAATAGAAGAGAACATTGAGGGCTTAGTAGGTATCGAGAATAACTCATTCCTTGAGGCACGTAACTTGTTTGATTCGGACCTATCCATCACTATGCCAGACGGACAGCAATTCTCAGTGAATGACCTAAGGGACTTCGATATGTTCCGCATCATGCCAGCGTATGACCGCCGTGTCAATGGTGACATCGCCATCATGGGGTCTACTGGTAAAACCACTAAGGAACTTAAg                                                                                                                                                                                                                                                                <  1:244581/301‑1 (MQ=255)`  
                                               `tGGTAGAGAAGTACGCTATGGATAAGGCTTATGGTATCTCCCACTCAGACCAGTTCACCAACAGTTCCATAATAGAAGAGAACATTGAGGGCTTAGTAGGTATCGAGAATAACTCATTCCTTGAGGCCCGTAACTTGTTTGATTCGGACCTATCCATCACTATGCCAGACGGACAGCAATTCTCAGTGAATGACCTAAGGAACTTCGATATGTTCCGCATCATGCCAGCGTATGACCGCCGTGTCAATGGTGACATCGCCATCATGGGGTCTACTGGTAAAACCACTAAAGAACTTAAGGa                                                                                                                                                                                                                                                              >  1:113148/1‑301 (MQ=255)`  
                                               `tGGTAGAGAAGTACGCTATGGATAAGGCTTATGGTATCTCCCACTCAGACCAGTTCACCAACAGTTCCATAATAGAAGAGAACATTGAGGGCTTAGTAGGTATCGAGAATAACTCATTCCTTGAGGCACGTAACTTGTTTGATTCGGACCTATCCATCACTATGCCAGACGGACAGCAATTCTCAGTGAATGACCTAAGGGACTTCGATATGTTCCGCATCATGCCAGCGTATGACCGCCGTGTCAATGGTGGCATCGCCATCATGGGGTCTACTGGTAAAACCACTaactgtcccttata                                                                                                                                                                                                                                                              >  1:68385/1‑289 (MQ=255)`  
                                               `tGGTAGAGAAGTACGCTATGGATAAGGCTTATGGTATCTCCCACTCAGACCAGTTCACCAACAGTTCCATAATAGAAGAGAACATTGAGGGCTTAGTAGGTATCGAGAATAACTCATTCCTTGAGGCACGTAACTTGTTTGATTCGGACCTATCCATCACTATGCCAGACGGACAGCAATTCTCAGTGAATGACCTAAGGGACTTCGATATGTTCCGCATCATGCCAGCGTATGACCGCCGTGTCAATGGTGACATCGCCATCATGGGGTCTACTGGTAAAACCACTAAGGAACTTAAggg                                                                                                                                                                                                                                                              >  1:503975/1‑300 (MQ=255)`  
                                                                  `ggATAAGGCTTATGGTATCTCCCACTCAGACCAGTTCACCAACAGTTCCATAATAGAAGAGAACATTGAGGGCTTAGTAGGTATCGAGAATAACTCATTCCTTGAGGCACGTAACTTGTTTGATTCGGACCTATCCATCACTATGCCAGACGGACAGCAATTCTCAGTGAATGACCTAAGGGACTTCGATATCTTCCGCATCATGCCAGCGTATGACCGCCGTGTCAATGGTGACATCGCCATCATGGGGTCTACTGGTAAAACCACTAAGGAACTTAAGGATGAGATTTTGGCTATCaaa                                                                                                                                                                                                                                           >  1:757027/1‑301 (MQ=255)`  
                                                                  `ggATAAGGCTTATGGTATCTCCCACTCAGACCAGTTCACCAACAGTTCCATAATAGAAGAGAACATTGAGGGCTTAGCAGGTATCGAGAATAACTCATTCCTTGAGGCACGTAACTTGTTTGATTCGGACCTATCCATCACTATGCCAGACGGACAGCAATTCTCAGTGATTGACCTAAGGGACTTCGATATGTTCCGCATCATGCCAGCGTATGACCGCCGTGTCAATGGTGACATCGCCATCATGGGGTCTACTGGTAAAACCACTAAGGAACTTAAGGATGAGATTTTGGCTCTCaaa                                                                                                                                                                                                                                           >  1:712080/1‑301 (MQ=255)`  
                                                                      `ccctcaatgttctcttctattatggaactgTTCACCTACAGTTCCATAATAGAAGAGAACATTGAGGGCTTAGTAGGTATCGAGAATAACTCATTCCTTGAGGCACGTAACTTGTTTGATTCGGACCTATCCATCACTATGCCAGACGGACAGCAATTCTCAGTGAATGACCTAAGGGACTTCGATATGTTCCGCATCATGCAAGCGTATGACCGCCGTGTCAATGGTGACATCGCCATCATGGGGTCTACTGGTAAAACCACTAAGGAACTTAATGATGAGATTTTGGCTCTCAAAGCGa                                                                                                                                                                                                                                       >  1:823397/30‑301 (MQ=255)`  
                                                                        `ggCTTATGGTATCTCCCACTCAGACCAGTTCACCAACAGTTCCATAATAGAAGAGAACATTGAGGGCTTAGTAGGTATCGAGAATAACTCATTCCTTGAGGCACGTAACTTGTTTGATTCGGACCTATCCATCACTATGCCAGACGGACAGCAATTCTCAGTGAATGACCTAAGGGACTTCGATATGTTCCGCATCATGCCAGCGTATGACCGCCGTGTCAATGGTGGCATCGCCATCATGGGGTCTACTGGTAAAACCACTAAGGAACTTAAGGATGAGATTTTGGCTCTCAAAGCGaag                                                                                                                                                                                                                                     >  1:213593/1‑300 (MQ=255)`  
                                                                        `ggCTTATGGTATCTCCCACTCAGACCAGTTCACCAACAGTTCCATAATAGAAGAGAACATTGAGGGCTTAGGAGGTATCGAGAATAACTCATTCCTTGAGGCACGTAACTTGTTTGATTCGGACCTATCCATCACTATGCCAGACGGACAGCAATTCTCAGTGAATGACCTAAGGGACTTCGATATGTTCCGCATCATGCCAGCGTATGACCGCCGTGTCAATGGTGACATCGCCATCATGGAGTCTACTGGTAAAACCACTAAGGAACTTAAGGATGAGATTTTTGcctgtctcttatac                                                                                                                                                                                                                                     >  1:556265/1‑288 (MQ=255)`  
                                                                                   `tctcCCACTCAGACCAGTTCACAAACAGTTCCATAATAGAAGAGAACATTGAGGACTTAGTAGGTATCGAGAATAACTCATTCCTTGAGGAACGTAACTTGTTTGATTCGGACCTATCCATCACTATGCCAGACGGACAGCAATTCTCAGTGAATGACCTAAGGGACTTCGATATGTTCCGCATCATGCCAGAGTATGACCGCCGTGTCAATGGTGACATCGCCATCATGGGGTCTACTGGTAAAACCACTAAGGAACTTAAGGATGAGATTTTGGCTCTCAAAGCGAAAGCTGAGGgagc                                                                                                                                                                                                                          <  1:355085/301‑2 (MQ=255)`  
                                                                                    `ctcCCACTCAGACCAGTTCACCAACAGTTCCATAATAGAAGAGAACATTGTGGGCTTAGTAGGTATCGAAAATAACTCATTCCTTGAGGCACTTAACTTGTTTTATTCGGACCTATCCATCACTATGCCAGACGGACAGCAATTCTCAGTGAATGACCTAAGGGACTTCTATATGTTCCGCATCATGCCAGCGTATGACCGCCGTGTCAATGGTGACATCGCCATCATGGGGTCTACTGGTAAAACCACTCAGGAACTTAAGGATGAGATTTTGGCTCTCAAAGCGAAAGCTGAGGgagag                                                                                                                                                                                                                         >  1:84284/1‑300 (MQ=255)`  
                                                                                    `ctcCCACTCAGACCAGTTCACCAACAGTTCCATAATAGAAGAGAACATTGAGGGCTTAGTAGGTATCGAGAATAACTCATTCCTTGAGGCACGTAACTTGTTTGATTCGGACCTATCCATCACTATGCCAGACGGACAGCAATTCTCAGTGAATGACCTAAGGGACTTCGATATGTTCCGCATCATGCCAGCGTATGACCGCCGTGTCAATGGTGACATCGCCATCATGGGGTCTACTGGTAAAACAACTAAGGAACTTACGGATGAGATTTTGGCTCTCAAAGCGAAAGCTGAGGGAGAc                                                                                                                                                                                                                         >  1:477962/1‑301 (MQ=255)`  
                                                                                    `ctcCCACTCAGACCAGTTCACCAACAGTTCCATAATAGAAGAGAACACTGAGGGCTTAGCAGGTATCGAGAATAACTCATTCCTTGAGGCACGTAACTTGTTTGATTCGGACCTATCCATCACTATGCCAGACGGACAGCAATTCTCAGTGAATGACCTAAGGGACTTCGATATGTTCCGCATCATGCCAGCGTATGACCGCCGTGTCAATGGTGACATCGCCATCATGGGGTCTACTGGTAAAACCACTAAGGAACTTAAGGATGAGATTTTGGCTCTCAAAGCGAAAGCTGAGGgagcc                                                                                                                                                                                                                         >  1:107026/1‑299 (MQ=255)`  
                                                                                    `ctcCCACTCAGACCAGTTCACCAACAGTTCCATAAGAGAAGAGAACATTGAGGGCTTAGTAGGTATCGAGAATAACTCATTCCTTGAGGCACGTAACTTGTTTGATTCGGACCTATCCATCACTATGCCAGACGGACAGCAATTCTCAGTGAATGACCTAAGGGACTTCGATATGTTCCGCATCATGCCAGCGTATGACCGCCGTGTCAATGGTGACATCGCCATCATGGGGTCTCCTGGTAAAACCACTAAGGAACTTAAGGATGAGATTTTGGCTCTCAAAGCGAAAGcctgtctctta                                                                                                                                                                                                                         >  1:386008/1‑291 (MQ=255)`  
                                                                                            `caatacAGTTCACCAATAGTTTCATAAGAGATGATAACATTGAGGTTTAAGATTGTATCGATAATATCTCATACCTTGAGCAGCGGATTTTGTTTGATTCGGACCGAACCACACCTATATCAGACGGACAGCAATGCTCAGTGGAAGGCCTAAGGGACTTCGATATTTTCCGCGTCATGGCAGCTTATGACCGCCGTGTCAATGGTGTCATCGCCATCATGGGGTCTACTGGTAAAACCAGTAAGGAACTTAAGGATGTGGTTTTGTCTCTCAAAGCGAAAGCTGAGGGAGACGGTaagaa                                                                                                                                                                                                                 <  1:298751/296‑1 (MQ=255)`  
                                                                                              `gggctcggagatgtgtataagagacaggaagaGAACATTGAGGGCTTAGTAGGTATCGAGAATAACTCATTCCTTGAGGCACGTAACTTGTTTGATTCGGACCTATCCATCACTATGCCAGACGGACAGCAATTCTCAGTGAATGACCTAAGGGACTTCGATATGTTCCGCATCATGCCAGCGTATGACCGCCGTGTCAATGGTGGCATCGCCATCATGGGGTCTACTGGTAAAACCACTAAGGAACTTAAGGATGAGATTTTGGCTCTCAAAGCGAAAGCTGAGGGAGACGGTAagaaga                                                                                                                                                                                                               <  1:404284/274‑1 (MQ=255)`  
                                                                                              `actcatttCACCAACAATTCCATAATAGAAGAGTACATTGAGGGCTTAGTAGGTATCGAGAATAACTCATTCCTTTGGGCACGTAACTTGTTTGATTCGGACCTATCCATCACTATGCCAGACGGACAGCAATTCTCAGTTCATGACCTAAGGGACTTCGATATGTTCCGCATCATGCCAGCGTATGACCGCCGTGTCAATGGTGACATCGCCATCATGGGGTCTACTGGTAAAACCACTAAGGAACTTAAGGATGAGATTTTGGCTCTCAAAGCGAAAGCTGAGGGAGACGGTAagaaga                                                                                                                                                                                                               <  1:497829/295‑1 (MQ=255)`  
                                                                                               `acaggTTCAACAACAGTTACATAATAGAAGAGAACATTGAGGGCTTAGTAGGTATCGAGAATAACTCATTCCTTGAGGCACGTAACTTGTTTGATTCGGACCTATCCATCACTATGCCAGACGGACAGCAATTCTCAGTGAATGACCTAAGGGACTTCGATATGTTCCGCATCATGCCAGCGTATGACCGCCGTGTCAATGGTGACATCGCCATCATGGGGTCTACTGGTAAAACCACTAAGGAACTTAAGGATGAGATTTTGGCTCTCAAAGCGAAAGCTGAGGGAGACGGTAAGAAGAc                                                                                                                                                                                                              <  1:62681/297‑1 (MQ=255)`  
                                                                                               `aCCAGGTCACCAACAGTTCCATAATAGAAGAGAACAGTGAGGGCTTAGTAGGTATCGAGAATAACTCATTCCTTGAGGCACGTAACTTGTTTGATTCGGACCTATCCATCACTATGCCAGACGGACAGCAATTCTCAGTGAATGACCTAAGGGACTTCGATATGTTCCGCATCATGCCAGCGTATGACCGCCGTGTCAATGGTGACATCGCCATCATGGGGTCTACTGGTAAAACCACTAAGGAACTTAAGGATGAGATTTTGGCTCTCAAAGCGAAAGCTGAGGGAGACGGTAAGAAGAc                                                                                                                                                                                                              <  1:784965/301‑1 (MQ=255)`  
                                                                                                `ccAGTTCACCAACAGTTCCATAATAGAAGAGAACATTGAGGGCTTAGTAGGTATCGAGAATAACTCATTCCTTGAGGCACGTAACTTGTTTGATTCGGACCTATCCATCACTATGCCAGACGGACAGCAATTCTCAGTGAATGACCTAAGGGACTTCGATATGTTCCGCATCATGCCAGCGTATGACCGCCGTGTCAATGGTGGCATCGCCATCATGGGGTCTACTGGTAAAACCACTAAGGAACTTAAGGATGAGATTTTGGCTCTCAAAGCGAAAGCTGAGGGAGACGGTAAGAAGACt                                                                                                                                                                                                             >  1:792547/1‑301 (MQ=255)`  
                                                                                                  `aGTTCACCAACAGTTCCATAATAGAAGAGAACATTGAGGGCTTAGTAGGTATCGAGAATAACTCATTCCTTGAGGCACGTAACTTGTTTGATTCGGACCTATCCATAACTATGCCAGACGGACAGCAATTCTCAGTGAATGACCTAAGGGACTTCGATATGTTCCGCATCATGCCAGCGTATGACCGCCGTGTCAATGGTGACATCGCCATCATGGGGTCTACTGGTAAAACCACTAAGGAACTTAAGGATGAGATTTTGGCTCTCAAAGCGAAAGCTGAGGGAGACGGTAAGAAGACTgg                                                                                                                                                                                                           <  1:579916/301‑1 (MQ=255)`  
                                                                                                   `gTTCACCAACAGTTCCATAATAGAAGAGAACATTGAGGGCTTAGTAGGTATCGAGAATAACTCATTCCTTGAGGCACGTAACTTGTTTGATTCGGACCTATCCATCACTATGCCAGACGGACAGCAATTCTCAGTGAATGACCTAAGGGACTTCGATATGTTCCGCATCATGCCAGCGTATGACCGCCGTGTCAATGGTGACATCGCCATCATGGGGTCTACTGGTAAAACCACTAAGGAACTTAATGATGAGATTTTGGCTCTCAAAGCGAAAGCTGAGGGAGACGGTAAGAAGACTGGc                                                                                                                                                                                                          >  1:433086/1‑301 (MQ=255)`  
                                                                                                   `gTTCACCAACAGTTCCATAATAGAAGAGAACATTGAGGGCTTAGTAGGTATCGAGAATAACTCATTCCTTGAGGCACGTAACTTGTTTGATTCGGACCTATCCATCACTATGCCAGACGGACAGCAATTCTCAGTGAATGACCTAAGGGACTTCGATATGTTCCGCATCATGCCAGCGTATGACCGCCGTGTCAATGGTGACATCGCCATCATGGGGTCTACTGGTAAAACCACTAAGGAACTTAAGGATGAGATTTTGGCTCTCCAAGCGAAAGCTGAGGGAGACGGTAAGAAGAcctgt                                                                                                                                                                                                          >  1:53465/1‑297 (MQ=255)`  
                                                                                                        `ccAACAGTTCCATAATAGAAGAGAACATTGAGGGCTTAGTAGGTATCGAGAATAACTCATTCCTTGAGGCACGTAACTTGTTTGATTCGGACCTATCCATCACTATGCCAGACGGACAGCAATTCTCAGTGAATGACCTAAGGGACTTCGATATGTTCCGCATCATGCCAGCGTATGACCGCCGTGTCAATGGTGGCATCGCCATCATGGGGTCTACTGGTAAAACCACTAAGGAACTTAAGGATGAGATTTTGGCTCTCAAAGCGAAAGCTGAGGGAGACGGTAAGAAGACTGGCGAGGt                                                                                                                                                                                                     <  1:721107/301‑1 (MQ=255)`  
                                                                                                          `acaggTTCCATAATAGAAGAGAACATTGAGGGCTTAGTAGGTATCGAGAATAACTCATTCCTTGAGGCACGTAACTTGTTTGAGTCGGACCTATCCATCACTATGCCAGACGGACAGCAATTCTCAGTGAATGACCTAAGGGACTTCGATATGTTCCGCATCATGCCAGCGTATGACCGCCGTGTCAATGGTGACATCGCCATCATGGGGTCTACTGGTAAAACCACTAAGGAACTTAATGATGAGATTTTGGCTCTCAAAGCGAAAGCTGAGGGAGACGGTAAGAAGACTGGCGAGGTAc                                                                                                                                                                                                   <  1:156150/297‑1 (MQ=255)`  
                                                                                                            `cAGTTCCATAATAGAAGAGAACATTGAGGGCTTAGTAGGTATCGAGAATAACTCATTCCTTGAGGCACGTAACTTGTTTGATTCGGACCTATCCATCACTATGCCAGACGGACAGCAATTCTCAGTGAATGACCTAAGGGACTTCGATATGTTCCGCATCATGCCAGCGTATGACCGCCGTGTCAATGGTGACATCGCCATCATGGGGTCTACTGGTAAAACCACTAAGGAACTTAAGGATGAGATTTTGGCTCTCAAAGCGAAAGCTGAGGGAGACGGTAAGAAGACTGGCGAGGTACAt                                                                                                                                                                                                 >  1:512642/1‑301 (MQ=255)`  
                                                                                                              `gTTCCATAATAGAAGAGAACATTGAGGGCTTAGTAGGTATCGAGAATAACTCATTCCTTGAGGCACGTAACTTGTTTGATTCGGACCTATCCATCACTATGCCAGACGGACAGCAATTCTCAGTGAATGACCTAAGGGACTTCGATATGTTCCGCATCATGCCAGCGTATGACCGCCGTGTCAATGGTGACATCGCCATCATGGGGTCTACTGGTAAAACCACTAAGGAACTTAAGGATGAGATTTTGGCTCTCAAAGCGAAAGCTGAGGGAGACGGTAAGAAGACTGGCGAGGTACATGc                                                                                                                                                                                               >  1:311868/1‑301 (MQ=255)`  
                                                                                                                 `ccATAATAGAAGAGAACATTGAGGGCTTAGTAGGTATCGAGAATAACTCATTCCTTGAGGCACGTAACTTGTTTGATTCGGACCTATCCATCACTATGCCAGACGGACAGCAATTCTCAGTGAATGACCTAAGGGACTTCGATATTTTCCGCATCATGCCAGCGTATGACCGCCGTGTCAATGGTGGCATCGCCATCATGGGGTCTACTGGTAAAACCACTAAGGAACTTAAGGATGAGATTTTGGCTCTCAAAGCGAAAGCTGAGGGAGACGGTAAGAAGACTGGCGAGGTACATGCttt                                                                                                                                                                                            >  1:306251/1‑301 (MQ=255)`  
                                                                                                                 `ccATAATAGAAGAGAACATTGAGGGCTTAGTAGGTATCGAGAATAACTCATTCCTTGAGGCACGTAACTTGTTTGATTCGGACCTATCCATCACTATGCCAGACGGACAGCAATTCTCAGTGAATGACCTAAGGGACTTCGATATGTTCCGCATCATGCCAGCGTATGACCGCTGTGTCAATGGTGACATCGCCATCATGGGGTCTACTGGTAAAACCACTAAGGAACTTAAGGATGAGATTTTGGCTCTCAAAGCGAAAGCTGAGGGAGACGGTAAGAAGACTGGCGAGGTACATGCttg                                                                                                                                                                                            >  1:589465/1‑300 (MQ=255)`  
                                                                                                                     `acaggaagaGAACATTGAGGGCTTAGTAGGTATCGAGAATAACTCATTCCTTGAGGCACGTAACTTGTTTGATTCGGACCTATCCATCACTATGCCAGACGGACAGCAATTCTCAGTGAATGACCTAAGGGACTTCGATATGTTCCGCATCATGCCAGCGTATGACCGCCGTGTCAATGGTGACATCGCCATCATAGTGTCTACTGGTAAAACCACTAAGGAACTTAAGGATGAGATTTTGGCTCTCAAAGCGAAAGCTGAGGGAGACGGTAAGAAGACTGGCGAGGTACATGCTTTAATg                                                                                                                                                                                        <  1:658596/297‑1 (MQ=255)`  
                                                                                                                      `ataGAAGAGAACATTGAGGGCTTAGTAGGTATCGAGAATAACTCATTCCTTGAGGCACGTAACTTGTTTGATTCGGACCTATCCATCACTATGCCAGACGGACAGCAATTCTCAGTGAATGACCTAAGGGACTTCGATATGTTCCGCATCATGCCAGCGTATGACCGCCGTGTCAATGGTGACATCGCCATCATGGGGTCTACTGGTAAAACCACTAAGGAACTTAATGATGAGATTTTGGCTCTCAAAGCGAAAGCTGAGGGAGACGGTAAGAAGACTGGCGAGGTACATGCTTTAATgg                                                                                                                                                                                       <  1:361905/301‑1 (MQ=255)`  
                                                                                                                      `ataGAAGAGAACATTGAGGGCTTAGTAGGTATCGAGAATAACTCATTCCTTGAGGCACGTAACTTGTTTGATTCGGACCTATCCATCACTATGCCAGACGGACAGCAATTCTCAGTGAATGACCTAAGGGACTTCGATATGTTCCGCATCATGCCAGCGTATGACCGCCGTGTCAATGGTGACATCGCCATCATGGGGTCTACTGGTAAAACCACTAAGGAACTTAATGATGAGATTTTGGCTCTCAAAGCGAAAGCTGAGGGAGACGGTAAGAAGACTGGCGAGGTACATGCTTTAATgg                                                                                                                                                                                       >  1:667824/1‑301 (MQ=255)`  
                                                                                                                        `agaagaGAACATTGAGGGCTTAGTAGGTATCGAGAATAACTCATTCCTTGAGGCACGTAACTTGTTTGATTCGGATCTATCCATCACTATGCCAGACGGACAGCAATTCTCAGTGAATGACCTAAGGGACTTCGGGATGTTCCGCATCATGCCAGCGTATGACCGCCGTGTCAGTGGTGGCATCGCCATCATGGGGTCTACTGGTAAAGCCACTAAGGAACTTAAGGATGAGATTTTGGCTCTCAAAGCGAAAGCTGATGGAGACGGTAAGAAGACTGGCGAGGTACATGCTTTAATGGAt                                                                                                                                                                                     <  1:92273/301‑1 (MQ=255)`  
                                                                                                                         `gaagaGAACATTGAGGGCTTAGTAGGTATCGAGAATAACTCATTCCTTGAGGCACGTAACTTGTTTTATTCGGACCTATCCATCACTATGCCAGACGGACAGCAATTCTCAGTGAATGACCTAAGGGACTTCGATATGTTCCGCATCATGCCAGCGTATGACCGCCGTGTCAATGGTGGCATCGCCATCATGGGGTCTACTGGTAAAACCACTAAGGAACTTAAGGATGAGATTTTGGCTCTCAAAGCGAAAGCTGAGGGAGACGGTAAGAAGACTGGCGAGGTACATACTTTAATGGATa                                                                                                                                                                                    >  1:799900/1‑301 (MQ=255)`  
                                                                                                                          `aagaGAACATTGAGGTCTTAGTAGGTATCGAGAATAACTCATTCCTTGAGGCACGTAACTTGTTTGATTCGGACCTATCCATCACTCTGCCAGACGGACAGCAATTCTCAGTGAATGACCTAAGGGACTTCGATATGTTCCGCATCATGCCAGCGTATGACCGCCGTGTCAATGGTGGCATCGCCATCATGGGGTCTACTGGTAAAACCACTAAGGAACTTAAGGATGAGATTTTGGCTCTCAAAGCGAAAGCTGAGGGAGACGGTAAGAAGACTGGCGAGGTACATGCTTTAATGGATAc                                                                                                                                                                                   <  1:748403/301‑1 (MQ=255)`  
                                                                                                                              `gaACATTGAGGGCTTAGTAGGTATCGAGAATAACTCATTCCTTGAGGCACGTAACTTGTTTGATTCGGACCTATCCATCACTATGCCAGACGGACAGCAATTCTCAGTGAATGACCTAAGGGACTTCGATATGTTCCGCATCATGCCAGCGTATGACCGCCGTGTCAATGGTGGCATCGCCATCATGGGGTCTACTGGTAAAACCACTAAGGAACTTAAGGATGAGATTTTGGCTCTCACAGCGAAAGCTGAGGGAGACGGTAAGAAGACTGGCGAGGTACATGCTTTAATGGATACCGtt                                                                                                                                                                               >  1:205792/1‑301 (MQ=255)`  
                                                                                                                                 `cATTGATGGCTTAGTAGGTATCGAGAATAACTCATTCCTTGAGGCACGTAACTTGTTTGATTCGGACCTATCCCTCTCTATGCCAGACTGTCAGCAATTCTCAGTCAATGACCTAATGTTCTTCGATATCTTCCGCATCATTCCTTCTTCTGACCGCCCTGTCAATGGTGACATCGCCATCCTTGTGTCTACTGGTAAATCCACTCATGAACTTAAGGATGCGTTTTTGTCTCTCAAAGCGAAAGCTGAGGGAGACGGTACGATGACTGTCTATGTACATGCTTTAAttgctgtctctttt                                                                                                                                                                            >  1:611457/1‑288 (MQ=255)`  
                                                                                                                                 `cATTGAGGGCTTAGTAGGTATCGAGAATAACTCATTCCTTGAGGCACGTAACTTGTTTGATTCGGACCTATCCATCACTATGCCAGACGGACAGCAATTCTCAGTGAATGACCTAAGGGACTTCGATATGTTCCGCATCATGCCAGCGTATGACCGCCGTGTCAATGGTGACATCGCCATCATGGGGTCTACTGGTAAAACCACTAAGGAACTTAATGATGAGATTTTGGCTCTCAAAGCGAAAGCTGAGGGAGACGGTAAGAAGACTGGCGAGGTACATGCTTTAATGGATACCATTAAg                                                                                                                                                                            >  1:228901/1‑301 (MQ=255)`  
                                                                                                                                  `aTTGAGGGCTTAGTAGGTATCGAGAATAACTCATTCCTTGAGGCACGTAACTTGTTTGATTCGGACCTATCCATCACTATGCCAGACGGACAGCAATTCTCAGTGAATGACCTAAGGGACTTCGATATGTTCCGCATCATGCCAGCGTATGACCGCCGTGTCAATGGTGACATCGCCATCATGGGGTCTACTGGTAAAACCACTAAGGAACTTAATGATGAGATTTTGGCTCTCAAAGCGAAAGCTGAGGGAGACGGTAAGAAGACTGGCGAGGTACATGCTTTAATGGATACCGTTAAGa                                                                                                                                                                           <  1:564905/301‑1 (MQ=255)`  
                                                                                                                                   `ttGAGGGCTTAGTAGGTATCGAGAATAACTCATTCCTTGAGGCACGTAACTTGTTTGATTCGGACCTATCCATCACTATGCCAGACGGACAGCAATTCTCAGTGAATGACCTAAGGGACTTCGATATGTTCCGCATCATGCCAGCGTATGACCGCCGTGTCAATGGTGACATCGCCATCATGGGGTCTACTGGTAAAACCACTAAGGAACTTAATGATGAGATTTTGGCTCTCAAAGCGAAAGCTGAGTGAGACGGTAAGAAGACTGGCGAGGTACATGCTTTAATGGATACCGTTAAGAt                                                                                                                                                                          <  1:572036/301‑1 (MQ=255)`  
                                                                                                                                         `gCTTAGTAGGTATCGAGAATAACTCATTCCTTGAGGCACGTAACTTGTTTGATTCGGACCTATCCATCACTATGCCAGACGGACAGCAATTCTCAGTGAATGACCTAAGGGACTTCGATATGTTCCGCATCATGCCAGCGTATGACCGCCGTGTCAATGGTGACATCGCCATCATGGGGTCTACTGGTAAAACCACTAAGGAACTTAATGATGAGATTTTGGCTCTCAAAGCGAAAGCTGAGGGAGACGGTAAGAAGACTGGCGAGGTACATGCTTTAATGGATACCGTTAAGATTCTTAc                                                                                                                                                                    >  1:449489/1‑301 (MQ=255)`  
                                                                                                                                          `cTTAGTAGGTATCGAGAATAACTCATTCCTTGAGGCACGTAACTTGTTTGATTCGGACCTATCCATCACTATGCCAGACGGACAGCAATTCTCAGTGAATGACCTAAGGGACTTCGATATGTTCCGCATCATGCCAGCGTATGACCGCCGTGTCAATGGTGACATCGCCATCATGGGGTCTACTGGTAAAACCACTAAGGAACTTAATGATGAGATTTTGGCTCTCAAGGCGAATGCTGAGGGAGACGGTAAGAAGACTGGCGAGGTACATGCTTTAATGGATACCGTTAAGATTCTTACt                                                                                                                                                                   >  1:74067/1‑301 (MQ=255)`  
                                                                                                                                            `tagcagGTATCGAGAATAACTCATTCCTTGAGGCACGTAACTTGTTTGATTCGGACCTATCCATCACTATGCCAGACGGACAGCAATTCTCAGTGAATGACCTAAGGGACTTCGATATGTTCCGCATCATGCCAGCGTATGACCGCCGTGTCAATGGTGACATCGCCATCATGGGGTCTACTGGTAAAACCACTAAGGAACTTAATGATGAGATTTTGGCTCTCAAAGCGAAAGCTGAGGGAGACGGTAAGAAGACTGGCGAGGTACATGCTTTAATGGATAcctgtctcttatacaaatc                                                                                                                                                                 >  1:456978/1‑284 (MQ=255)`  
                                                                                                                                             `agtagGTATCGAGAATAACTCATTCCTTGAGGCACGTAACTTGTTTGATTCGGACCTATCCATCACTATGCCAGACGGACAGCAATTCTCAGTGAATGACCTAAGGGACTTCGATATGTTCCGCATCATGCCAGCGTCTGACCGCCGTGTCAATGGTGGCATCGCCATCATGGGGTCTACTGGTAAAACCACTAAGGAACTTAAGGATGAGATTTTGGCTCTCAAAGCGAAAGCTGAGGGAGACGGTAAGAAGACTGGCGAGGTACATGCTTTAATgctgtctcttatacacatctccgag                                                                                                                                                                >  1:335731/1‑277 (MQ=255)`  
                                                                                                                                                         `gaATAACTCATTCCTTGAGGCACGTAACTTGTTTGATTCGGCCCTATCCATCACTATGCCAGACGGACAGCAATTCTCAGTGAATGACCTAAGGGACTTCGATATGTTCCGCATCATGCCAGCGTATGACCGCCGTGTCAATGGTGGCATCGCCATCATGGGGTCTACTGGTAAAACCACTAAGGAACTTAAGGATGAGATTTTGGCTCTCAAAGCGAAAGCTGAGGGAGACGGTCAGCAGACTGGCGAGGTACATACTTTAATGGATACCGTTAATATTCTTACTGGTCGTGCTAGACGc                                                                                                                                                    >  1:54993/1‑301 (MQ=255)`  
                                                                                                                                                         `gaATAACTCATTCCTTGAGGCACGTAACTTGTTTGATTCGGACCTATCCATCACTATGCCAGACGGACAGCAATTCTCAGTGAATGACCTAAGGGACTTCGATATGTTCCGCATCATGCCAGCGTATGACCGCCGTGTCAATGGTGACATCGCCATCATGGGGTCTACTGGTAAAACCACTAAGGAACTTAATGATGAGATTTTGGCTCTCAAAGCGAAAGCTGAGGGAGACGGTAAGAAGACTGGCGAGGTACATGCTTTAATTGATACCGTTAAGATTCTTACTGGTCGTGCTAGACGc                                                                                                                                                    >  1:691143/1‑301 (MQ=255)`  
                                                                                                                                                                `acagtCCTTGAGGCACGTAACTTGTTTGATTCGGACCTATCCATCACTATGCCAGACGGACAGCAATTCTCAGTGAATGACCTAAGGGACTTCGATATGTTCCGCATCATGCCAGCGTATGACCGCCGTGTCAATGGTAACATCGCCATCATGGGGTCTACTGGTAAAACCACTAAGGAAATTAAGGATGAGATTTTGGCTCTCAAAGCGAAAGCTGAGGGAGACGGTAAGAAGACTGGCGAGGTACATGCTTTAATGGATACCGTTAAGATTCTTACTGGTCGTGCTAGACGCAATCAgg                                                                                                                                             <  1:770271/297‑1 (MQ=255)`  
                                                                                                                                                                       `ttGAGGCACGTAACTTGTTTGATTCGGACCTATCCATCACTATGCCAGACGGACAGCAATTCTCAGTGAATGACCTAAGGGACTTCGATATGTTCCGCATCATGCCAGCGTATGACCGCCGTGTCAATGGTGGCATCGCCATCATGGGGTCTACTGGTAAAACCACTAAGGAACTTAAGGATGAGATTTTGGCTCTCAAAGCGAGAGCTGAGGGAGACGGTAAGAAGACTGGCGAGGTACATGCTTTAATGGATACCGTTAAGATTCTTACTGGTCGTGCTAGACGCAATCAGGACACtgt                                                                                                                                      <  1:282875/301‑1 (MQ=255)`  
                                                                                                                                                                           `ggCACGTAACTTGTTTGATTCGGACCTATCCATCACTATGCCAGACGGACAGCAATTCTCAGTGAATGACCTAAGGGACTTCGATATGTTCCGCATCATGCCAGCGTATGACCGCCGTGTCAATGGTGACATCGCCATCATGGGGTCTACTGGTAAAACCACTAAGGAACTTAAGGATGAGATTTGGGCTCTCAAAGCGAAAGCTGAGGGAGACGGTAAGAAGACTGGCGAGGTACATGCTTTAATGGATACCGTTAAGATTCTTACTGGTCGTTCTAGACGCAATCAGGACACTGTTTgg                                                                                                                                  >  1:619928/1‑301 (MQ=255)`  
                                                                                                                                                                            `gCACGTCACTTGTTTGATTCGGACCTATCCATCACTATGCCAGACGGACAGCAATTCTCAGTGAATGACCTAAGGGACTTCGATATGTTCCGCATCATGCCAGCGTATGACCGCCGTGTCAATGGTGACATCGCCAGCATGGGGTCTACTGGTAAAACCACTAAGGAACTTAAGGATGAGATTTTGTCTCTCAAAGCGAAAGCTGAGGGAGACGGTAAGAAGACTGGCGAGGTACATGCTTTAATGGATACCGTTAAGATTCTTACTGGTCGTGCTAGACGCAATCAGGACACTGTGTggg                                                                                                                                 <  1:347994/301‑1 (MQ=255)`  
                                                                                                                                                                            `gCACGTAACTTGTTTGATTCGGACCTATCCATCACTATGTCAGACGGACAGCAATTCTCAGTGAATGACCTAAGGGACTTCGATATGTTCCGCATCATGCCAGCGTATGACCGCCGTGTCAATGGTGGCATCGCCATCATGGGGTCTATTGGTAAAACCACTAAGGAACTTAAGGATGAGATTTTGGCTCTCAAAGCGAAAGCTGAGGGAGACGGTAAGAAGACTGGCGAGGTACATGCTTTAATGGATACCGTTAAGATTCTTACTGGTCGTGCTAGACGCAATCAGGACACTGTGTggg                                                                                                                                 <  1:12843/301‑1 (MQ=255)`  
                                                                                                                                                                              `ggagatgtgtataagagacagccTATCCATCACTATGCCAGACGGACAGCAATTCTCAGTGAATGACCTAAGGGACTTCGATATGTTCCGCATCATGCCAGCGTATGACCGCTGTGTCAATGGTGACATCGCCATCATGGGGTCTACTGGTAAAACCACTAAGGAACTTAAGGATGAGATTTTGGCTCTCAAAGCGAAAGCTGAGGGAGACGGTAAGAAGACTGGCGAGGTACATGCTTTAATGGATACCGTTAAGATTCTTACTGGTCGTGCTAGACGCAATCAGGACACTGTGTGGGaa                                                                                                                               <  1:705236/280‑1 (MQ=255)`  
                                                                                                                                                                              `ccGTAACTTGTTTGATTCGGACCTATCCATCACTATGCCAGACGGACAGCAATTCTCAGTGAATGACCTAAGGGACTTCGATATGTTCCGCATCATGCCAGCGTATGACCGCCGTGTCAATGGTGACATCGCCATCATGGGGTCTACTGGTAAAACCACTAAGGAACTTAAGGATGAGATTTGGGCTCTCAAAGCGAAAGCTGAGGGAGTCGGTAAGAAGACTGGCGAGGTACATGCTTTAATGGATACCGTTAAGATTCTTACTGGTCGTGCTAGACGCAATCAGGACACTGTGTGGGaa                                                                                                                               <  1:727926/300‑1 (MQ=255)`  
                                                                                                                                                                              `aCGTAACTTGTTTGATTCGGACCTATCCATCACTATGCCAGACGGACAGCAATTCTCAGTGAATGACCTAAGGGACTTCGATATGTTCCGCATCATGCCAGCGTATGACCGCCGTGTCAATGGTGACATCGCCATCATGGGGTCTACTGGTAAAACCACTAAGGAACTTAATGATGAGATTTTGGCTCTCAAAGCGAAAGCTGAGGGAGACGGTAAGAAGACTGGCGAGGTACATGCTTTAATGGATACCGTTAAGATTCTTACTGGTCGTGCTAGACGCAATCAGGACACTGTGTGGGaa                                                                                                                               <  1:425038/301‑1 (MQ=255)`  
                                                                                                                                                                              `aCGTAACTTGTTTGATTCGGACCTATCCATCACTATGCCAGACGGACAGCAATTCTCAGTGAATGACCTAAGGGACTTCGATATGTTCCGCATCATGCCAGCGTATGACCGCCGTGTCAATGGTGACATCGCCATCATGGGGTCTACTGGTAAAACCACTAAGGAACTTAAGGATGAGATTTTGGCTCTCAAAGCGAAAGCTGAGGGAGACGGTAAGAAGACTGGCGAGGTACATGCTTTAATGGATACCGTTAAGATTCTTACTGGTCGTGCTAGACGCAATCAGGACACTGTGTGGGaa                                                                                                                               <  1:164456/301‑1 (MQ=255)`  
                                                                                                                                                                                      `tGTTTGATTCGGACCTATCCATCACTATGTCAGACGGACAGCAATTCTCAGTGAATGACCTAAGGGACTTCGATATGTTCCGCATCATGCCAGCGTATGACCGCCGTGTCAATGGTGACATCGCCATCATGGGGTCTACTGGTAAAACCACTAAGGAACTTAAGGATGAGATTTTGGCTCTCAAAGCGAAAGCTGAGGGAGACGGTAAGAAGACTGGCGAGGTACATGCTTTAATGGATACCGTTAAGATTCTTACTGGTCGTGCTAGACGCAATCAGGACACTGTGTGGGaactgtctct                                                                                                                       >  1:368691/1‑293 (MQ=255)`  
                                                                                                                                                                                       `gTTTGATTCGGACCTATCCATCACTATGCCAGACGGACAGCAATTCTCAGTTAATGACCTAAGGGACTTCGATATGTTCCGCATCATGCCAGCGTATGACCGCCGTGTCAATGGTGACATCGCCATCATGGGGTCTACTGGTAAAACCACTAAGGAACTTAAGGATGAGATTTTGGCTCTCAAAGCGAAAGCTGAGGGAGACGGTAAGAAGACTGGCGCGGTACATGCTTTAATGGATACCGTTAAGATTCTTACTGGTCGTGCTAGACGCAATCAGGACACTGTGTGGGAAACCTCAccg                                                                                                                      >  1:26133/1‑299 (MQ=255)`  
                                                                                                                                                                                            `aTTCGGACCTATCCATCACTATGCCAGACGGACAGCAATTCTCAGTGAATGACCTAAGGGACTTCGATATGTTCCGCATCATGCCAGCGTATGACCGCCGTGTCAATGGTGACATCGCCATCATGGGGTCTACTGGTAAAACCACTAAGGAACTTAAGGATGAGATTTTGGCTCTCAAAGCGAAAGCTGAGGGAGACGGTAAGAAGACTGGCGAGGTACATGCTTTAATGGTTACAGTTAAGATTCTTACTGGTCGTGCTAGACGCAATCAGGACACTGTGTGGGAAACCTCACTGCGTGc                                                                                                                 >  1:676861/1‑301 (MQ=255)`  
                                                                                                                                                                                            `aTTCGGACCTATCCATCACTATGCCAGACGGACAGCAATTCTCAGTGAATGACCTAAGGGACTTCGATATGTTCCGCATCATGCCAGCGTATGACCGCCGTGTCAATGGTGACATCGCCATCATGGGGTCTACTGGTAAAACCACTAAGGAACTTAAGGATGAGATTTTGGCTCTCAAAGCGAAAGCTGAGGGAGACGGTAAGAAGACTGGCGAGGTACATGCTTTAATGGATACCGTTAAGATTCTTACTGGTCGTGCTAGACGCAATCAGGACACTGTGTGGGAAACCTCACTGCGTGc                                                                                                                 >  1:808071/1‑301 (MQ=255)`  
                                                                                                                                                                                                    `cTATCCATCACTATGCCAGACGGACAGCAATTCTCAGTGAATGACCTAAGGGACTTCGATATGTTCCGCATCATGCCAGCGTATGACCGCCGTGTCAATTGTGACATCGCCATCATGGGGTCTACTGGTAAAACCACTAAGGAACTTAATGATGAGATTTTGGCTCTCAAAGCGAAAGCTGAGGGAGACGGTAAGAAGACTGGCGAGGTACATGCTTTAATGGATACCGTTAAGATTCTTACTGGTCGTGCTAGACGCAATCAGGACACTGTGTGGGAAACCTCACTGCGTGCCATCAATg                                                                                                         >  1:734981/1‑301 (MQ=255)`  
                                                                                                                                                                                                    `cTATCCATCACTATGCCAGACGGACAGCAATTCTCAGTGAATGACCTAAGGGACTTCGATATGTTCCGCATCATGCCAGCGTATGACCGCCGTGTCAATGGTGACATCGCCATCATGGGGTCTACTGGTAAAACCACTAAGGAACTTAAGGATGAGATTTTGGCTCTCAAAGCGAAAGCTGAGGGAGACGGTAAGAAGACTGGCGAGGTACATGCTTTAATGGATACCGTTAAGATTCTTACTGGTCGTGCTAGACGCAATCAGGACACTGTGTGGGAAACCTCACTGCGTGCCATCAATg                                                                                                         <  1:290498/301‑1 (MQ=255)`  
                                                                                                                                                                                                      `aTCCATCACTATGCCAGACGGACAGCAATTCTCAGTGAATGACCTAAGGGACTTCGATATGTTCCGCATCATGCCAGCGTATGACCGCCGTGTCAATGGTGGCATCGCCATCATGGGGTTTACTGGTAAAACCACTAAGGAACTTAAGGATGAGATTTTGGCTCTCAAAGCGAAAGCTGAGGGAGACGGTAAGAAGACTGGCGAGGTCCATGCTTTAATGGAAACCGTTAAGATTCTTATTGTTCTTGCTAAACGCACTCAGGACACTGGGTGGGAAACCTCAcgggttgcactcattgac                                                                                                       >  1:41748/1‑284 (MQ=255)`  
                                                                                                                                                                                                      `aTCCATCACTATGCCAGACGGACAGCAATTCTCAGTGAATGACCTAAGGGACTTCGATATGTTCCGCATCATGCCAGCGTATGACCGCCGTGTCAATGGTGGCATCGCCATCATGGGGTCTACTGGTAAAACCACTAAGGAACTTAAGGATGAGATTTTGGCTCTCAAAGCGAAAGCTGAGGGAGACGGTAAGAAGACTGGCGAGGTACATGCTTTAATGGATACCGTTAAGATTCTTACTGGTCGTGCTAGACGCAATCAGGACACTGTGTGGGAAACCTCACTGCGTGCCATCAATGAc                                                                                                       >  1:6975/1‑301 (MQ=255)`  
                                                                                                                                                                                                      `aTCCATCACTATGCCAGACGGACAGCAATTCTCAGTGAATGACCTAAGGGACTTCGATATGTTCCGCATCATGCCAGCGTATGACCGCCGTGTCAATGGTGACATCGCCATCATGGGGTCTACTGGTAAAACCACTAAGGAACTTAATGATGAGATTTTGGCTCTCAAAGCGAAAGCTGAGGGAGACGGTAAGAAGACTGGCGAGGTACATGCTTTAATGGATACCGTTAAGATTCTTACTGGTCGTGCTAGACGCAATCAGGACACTGTGTGGGAAACCTCACTGCGTGCCATCAATGAc                                                                                                       >  1:120944/1‑301 (MQ=255)`  
                                                                                                                                                                                                        `ccATCACTATGCCAGACGGACAGCAATTCTCAGTGAATGACCTAAGGGACTTCGATATGTTCCGCATCATGCCAGCGTATGACCGCCGTGTCAATGGTGACATCGCCATCATGGTGTCTACTGGTAAAACCACTAAGGAACTTAAGGATGAGATTTTGGCTCTCAAAGCGAAAGCTGAGGGAGACGGTAAGAAGACTGGCGAGGTACATGCTTTAATGGATACCGTTAAGATTCTTACTGGTCGTGCTAGACGCAATCAGGACACTGTGTGGGAAACCTCACTGCGTGCCATCAATGACCt                                                                                                     <  1:14201/301‑1 (MQ=255)`  
                                                                                                                                                                                                        `ccATCACTATGCCAGACGGACAGCAATTCTCAGTGAATGACCTAAGGGACTTCGATATGTTCCGCATCATGCCAGCGTATGACCGCCGTGTCAATGGTGACATCGCCATAATGGGGTCTACTGGTAAAACCACTAAGGAACTTAAGGATGAGATTTTGGCTCTCAAAGCGAAAGCTGAGGGAGACGGTAAGAAGACTGGCGAGGTACATGCTTTAATGGATACCGTTAAGATTCTTACTGGTAGTGCTAGACGCAATCAGGAAACTGTGTGGGAACCCTCACTTCGTGCCATCAATgcact                                                                                                     >  1:410212/1‑297 (MQ=255)`  
                                                                                                                                                                                                        `ccAACAATATCCCAGACGGACAGCAATTCTCAGTCAATGACCTAAGAGACTTCGATACGTTCCGCATCATGCCAGCGTATGACCACCGTGTCAATGGTGACATCGCCATCATGGGGTCTACTGGTAAAACCACTAAGGCACTTAAGGATGAGATTTTGGCTCTCAAAGCGAAAGCTGAGGGAGACGGTAAGAAGACTGGCGAGGTACATGCTTTAATGGATACCGTTAAGATTCTTACTGGTCGTGCTAGACGCAATCAGGACACTGTGTGGGAAACCTCACTGCGTGCCATCAATGACCt                                                                                                     <  1:596568/301‑1 (MQ=255)`  
                                                                                                                                                                                                           `tCACTATGCCAGACGGACAGCAATTCTCAGTGAATGACCTAAGGGACTTCGATATGTTCCGCATCATGCCAGCGTATGACCGCCGTGTCAATGGTGACATCGCCATCATGGGGTCTACTGGTAAAACCACTAAGGAACTTAATGATGAGATTTTGGCTCTCAAAGCGAAAGCTGAGGGAGACGGTAAGAAGACTGGCGAGGTACATGCTTTAATGGATACCGTTAAGATTCTTACTGGTCGTGCTAGACGCAATCAGGACACTGTGTGGGAAACCTCACTGCGTGCCATCAATGACCTAgg                                                                                                  >  1:483981/1‑301 (MQ=255)`  
                                                                                                                                                                                                            `cACTATGCCAGACGGACAGCAATTCTCAGTGAATGACCTAAGGGACTTCGATATGTTCCGCATCATGCCAGCGTATGACCGCCGTGTCAATGGTGGCATCGCCATCATGGGGTCTACTGGTAAAACCACTAAGGAACTTAAGGATGAGATTTTGGCTCTCAAAGCGAAAGCTGAGGGAGACGGTAAGAAGACTGGCGAGGTACATGCTTTAATGGATACCGTTAAGATTCTTACTGGTCGTGCTAGACGCAATCAGGACACTGTGTGGGAAACCTCACTGCGTGCCATCAATGACCTAggg                                                                                                 <  1:190918/301‑1 (MQ=255)`  
                                                                                                                                                                                                             `aCTATGCCAGACGGACAGCAATTCTCAGTGAATGACCTAAGGGACTTCGATATGTTCCGCATCATGTCAGCGTATGGCCGCCGTGTCAATGGTGACATCGCCATCATGGGGTCTACTGGTAAAACCACTAAGGAACTTAATGATGAGATTTTGGCTCTCAAAGCGAAAGCTGAGGGAGACGGTAAGAAGACTGGCGAGGTACATGCTTTAATGGATACCGTTAAGATTCTTACTGGTCGTGCTAGACGCAATCAGGAAGCTGTGTGGGAAACCTCACTGCGTGCCATCAATGACCTAGGGt                                                                                                <  1:268940/301‑1 (MQ=255)`  
                                                                                                                                                                                                                `aTGCCAGACGGACAGCAATTCTCAGTGAATGACCTAAGGGACTTCGATATGTTCCGCATCATGCCAGCGTATGACCGCCGTGTCAATGGTGACATCGCCATCATGGGGTCTACTGGTAAAACCACTAAGGAACTTAATGATGAGATTTTGGCTCTCAAAGCGAAAGCTGAGGGAGACGGTAAGAAGACTGGCGAGGTACATGCTTTAATGGATACCGTTAAGATTCTTACTGGTCGTGCTAGACGCAATCAGGACACTGTGTGGGAAACCTCACTGCGTGCCATCAATGACCTAGGGttct                                                                                             >  1:816342/1‑301 (MQ=255)`  
                                                                                                                                                                                                                `aTGCCAGACGGACAGCAATTCTCAGTGAATGACCTAAGGGACTTCGATATGTTCCGCATCATGCCAGCGTATGACCGCCGTGTCAATGGTGACATCGCCATCATGGGGTCTACTGGTAAAACCACTAAGGAACTTAAGGATGAGATTTTGGCTCTCAAAGCGAAAGCTGAGGGAGACGGTAAGAAGACTGGCGAGGTACATGCTTTAATGGATACCGTTAAGATTCTTACTGGTCGTGCTAGACGCAATCAGGACACTGTGTGGGAAACCTCACTGCGTGCCATCAATGACCTAGGGttct                                                                                             >  1:603475/1‑301 (MQ=255)`  
                                                                                                                                                                                                                                    `atgagagacagACCTAAGGGACTTCGATATGTTCCGCATCATGCCAGCGTATGACCGCCGTGTCAATGGTGACATCGCCATCATGGGGTCTACTGGTAAAACCACTAAGGAACTTAAGGATGAGATTTTGGCTCTCAAAGCGAAAGCTGAGGTAGACGGTAAGAAGACTTGCGAGGTACATGCTTTAATGGATACCGTTAAGATTCTTACTGGTCATGCTAGACGCAATCGGGACACTGTGTGGGAAACCTCACTGCGTGCCATCAATGACCTAGGGTTCTTCGCTAAGAACGCCTACATg                                                                         <  1:640795/291‑1 (MQ=255)`  
                                                                                                                                                                                                                                     `tcAGTGAATGACCTAAGGGACTTCGATATGTTCCGCATCATGCCAGCGTATGACCGCCGTGTCAATGGTGGCATCGCCATCATGGGGTCTACTGGTAAAACCACTAAGGAACTTAAGGATGAGATTTTGGCTCTCAAAGCGAAAGCTGAGGGAGACGGTAAGAAGACTGGCGAGGTACATGCTTTAATGGATACCGTTAAGATTCTTACTGGTCGTGCTAGACGCAATCAGGAAACTGTGTGGGAAACCTCACTGCGTGCCATCAATGACCTAGGGTTCTTCGCTAAGAACGCCTACATgg                                                                        <  1:541926/301‑1 (MQ=255)`  
                                                                                                                                                                                                                                     `gcAGTGAAAGACCTAAGGGACTTCGATATGTTCCGCATCATGCCAGCGTATGACCGCCGTGTCAATGGTGACATCGCCATCATGGGGTCTACTGGTAAAACCACTAAGGAACTTAATGATGAGATTTTGGCTCTCAAAGCGAAAGCTGAGGGAGACGGTAAGAAGACTGGCGAGGTACATGCTTTAATGGATACCGTTAAGATTCTTACTGGTCGTGCTAGACGCAATCAGGACGCTGTGTGGGAAACCTCACTGCGTGCCATCAATGACCTAGGGTTCTTCGCTAAGAACGCCTACATgg                                                                        <  1:634488/300‑1 (MQ=255)`  
                                                                                                                                                                                                                                      `cAGTGAATGACCTAAGGGACTTCGATATGTTCCGCATCATGCCAGCGTATGACCGCCGTGTCAATGGTAACATCGCCATCATGGGGTCTACTGGTAAAACCACTAAGGAACTTAAGGATGAGATTTTGGCTCTCAAAGCGAAAGCTGAGGGAGACGGTAAGAAGACTGGCGAGGTACATGCTTTAATGGATACCGTTAAGATTCTTACTGGTCGTGCTAGACGCAATCAGGACACTGTGTGGGAAACCTCACTGCGTGCCATCAATGACCTAGGGTTCTTCGCTAAGAACGCCTACATggg                                                                       <  1:649497/301‑1 (MQ=255)`  
                                                                                                                                                                                                                                        `gTGAATGACCTAAGGGACTTCGATATGTTCCGCATCATGCCAGCGTATGACCGCCGTGTCAATGGTGACATCGCCATCATGGGGTCTACTGGTAAAACCACTAAGGAACTTAATGATGAGATTTTGGCTCTCAAAGCGAAAGCTGAGGGAGACGGTAAGAAGACTGGCGAGGTACATGCTTTAATGGATACCGTTAAGATTCTTACTGGTCGTGCTAGACGCAATCAGGACACTGTGTGGGAAACCTCACTGCGTGCCATCAATGACCTAGGGtctgtctcttatacacatctccgagccc                                                                     >  1:171772/1‑274 (MQ=255)`  
                                                                                                                                                                                                                                               `aCCTAAGGGACTTCGATATGTTCCGCATCATGCCATCGTATGACCGCCGTGTCAATGGTGACATCGCCATCATGGGGTCTACTGGTAAAACCACTAAGGAACTTAAGGATGAGATTTTGGCTCTCAAAGCGAAAGCTGAGGGAGACGGTAAGAAGACTGGCGAGGTACATGCTTTAATGGATACCGTTAAGATTCTTACTGGTCGTCCTAGACGCAATCAGGACACTGTGTGAGAAACCTCACTGCGTGCCATCAATGACCTAGGGTTCTTCGCTAAGAACGCCTACATGGGTACTCAgct                                                              >  1:181474/1‑299 (MQ=255)`  
                                                                                                                                                                                                                                                `ccTAAGGGACTTCGATATGTTCCGCATCATGCCAGCGTATGACCGCCGTGTCAATGGTGGCATCGCCATCATGGGGTCTACTGGTAAAACCACTAAGGAACTTAAGGATGAGATTTTGGCTCTCAAAGCGAAAGCTGAGGGAGACGGTAAGAAGACTGGCGATGTACATGCTTTAATGGATACCGTTAAGATTCTTACTGGTCGTGCTAGACGCAATCAGGACACTGTGTGGGAAACCTCACTGCGTGCCATCAATGACCTAGGGTTCTTAGCTAAGAACGCCTACATGGGTGCTCAGAAc                                                             >  1:221529/1‑301 (MQ=255)`  
                                                                                                                                                                                                                                                `ccTAAGGGACTTCGATATGTTCCGCATCATGCCAGCGTATGACCGCCGTGTCAATGGTGACATCGACATCATGGGGTCTACTGGTAAAACCACTAAGGAACTTAAGGATGAGATTTTGGCTCTCAAAGCGAAAGCTGAGGGAGACGGTAAGAAGACTGGCGAGGTACATGCTTTAATGGATACCGTTAAGATTCTTACTGGTCGTGCTAGACGCAATCAGGACACTGTGTGGGAAACCTCACTGCGTGCCATCAATGACCTAGGGTTCTTCGCTAAGAACGCCTACATGGGTGCTCAGAAc                                                             <  1:469116/301‑1 (MQ=255)`  
                                                                                                                                                                                                                                                     `gggACTTCGATATGTTCCGCATCAGGCCAGCGTATGACCGCCGTGTCAATGGTGACATCGCCATCATGGGGTCTACTGGTAAAACCACTAAGGAACTTAATGATGAGATTTTGGCTCTCAAAGCGAAAGCTGAGGGAGACGGTAAGAAGACTGGTGAGGTACATGCTTTAATGGATACCGTTAAGATTCTTACTGGTCGTGCTAGACGCAATCAGGACACTGTGTGGGAAACCTCACTGCGTGCCATCAATGACCTAGGGTTCTTCGCTAAGAACGCCTACATGGGTGCTCAGAACATTAc                                                        <  1:377687/301‑1 (MQ=255)`  
                                                                                                                                                                                                                                                       `agcTTCGATATGTTCCGCATCATGCCAGCGTATGACCGCCGTGTCAATGGTGGCATCGCCATCATGGGGTCTACTGGTAAAACCACTAAGGAACTTAAGGATGAGATTTTGGCTCTCAAAGCGAAAGCTGAGGGAGACGGTAAGAAGACTGGCGAGGTACATGCTTTAATGGATACCGTTAAGATTCTTACTGGTCGTGCTAGACGCAATCAGGACACTGTGTGGGAAACCTCACTGCGTGCCATCAATGACCTAGGGTTCTTCGCTAAGAACGCCTACATGGGTGCTCAGAACATTACgg                                                      <  1:135863/299‑1 (MQ=255)`  
                                                                                                                                                                                                                                                       `aagagacagatGTTCCGCATCATGCCAGCGTATGACCGCCGTGTCAATGGTGACATCGCCATCATGGGGTCTACTGGTAAAACCACTAAGGAACTTAAGGATGAGATTTTGGCTCTCAAAGCGAAAGCTGAGGGAGACGGTAAGAAGACTGGCGAGGTACATGCTTTAATGGATACCGTTAAGATTCTTACTGGTCGTGCTAGACGCAATCAGGACACTGTGTGGGAAACCTCACTGCGTGCCATCAATGACCTAGGGTTCTTCGATAAGAACGCCTACATGGGTGCTCAGAACATTACgg                                                      <  1:396937/292‑1 (MQ=255)`  
                                                                                                                                                                                                                                                        `aCTTCGATATGTTCCGCATCATGCCAGCGTATGACCGCTGTGTCAATGGTGACATCGCCATCATGGGGTCTACTGGTAAAACCACTAAGGAACTTAATGATGAGATTTTGGCTCTCAAAGCGAAAGCTGAGGGAGACGGTAAGAAGACTGGCGAGGTACATGCTTTAATGGATACCGTTAAGATTCTTACTGGTCGTGCTAGACGCAATCAGGACACTGTGTGGGAAACCTCACTGCGTGCCATCAATGACCTAGGGTTCTTCGCTAAGAACGCCTACATGGGTGCTCAGAACATTACGga                                                     >  1:149054/1‑301 (MQ=255)`  
                                                                                                                                                                                                                                                             `gATATGTTCCGCATCATGCCAGCGTATGACCGCCGTGTCAATGGTTACATCGCCATCATGGGGTCTACTGGTAAAACCACTAAGGAACTTAAGGATGAGATTTTGGCTCTCAAAGCGAAAGCTGAGGGAGACGGTAAGAAGACTGGCGAGGTACATGCTTTAATGGATACCGTTAAGATTCTTACTGGTCGTGCTAGACGCAATCAGGACACTGTGTGGGAAACCTCACTGCGTGCCATCAATGACCTAGGGTTCTTCGCTAAGAACGCCTACATGGGTGCTCAGAACATTACGGAGAttt                                                >  1:25211/1‑300 (MQ=255)`  
                                                                                                                                                                                                                                                                 `ggTTCCGCATCATGCCAGCGTATGACCGCCGTGTCAATGGTGGCATCGCCATCATGGGGTCTACTGGTAAAACCACTAAGGAACTTAAGGATGAGATTTTGGCTCTCAAAGCGAAAGCTGAGGGAGACGGTAAGAAGACTGGCGAGGTACATGCTTTAATGGATACCGTTAAGATTCTTACTGGTCGTGCTAGACGCAATCAGGACACTGTGTGGGAAACCTCACTGCGTGCCATCAATGACCTAGGGTTCTTCGCTAAGAACGCCTACATGGGTGCTCAGAACATTAcctgtctcttata                                            >  1:490927/2‑289 (MQ=255)`  
                                                                                                                                                                                                                                                                  `gTTCCGCATCATGCCAGCGTATGACCGCCGTGTCAATGGTGACATCGCCATCATGGGGTCTACTGGTAAAACCACTAAGGAACTTAATGATGAGATTTTGGCTCTCAAAGCGAAAGCTGAGGGAGACGGTAAGAAGACTGGCGAGGTACATGCTTTAATGGATACCGTTAAGATTCTTACTGGTCGTGCTAGACGCAATCAGGACACTGTGTGGGAAACCTCACTGCGTGCCATCAATGACCTAGGGTTCTTCGCTAAGAACGCCTACATGGGTGCTCAGAACATTCCGGAGATTGCCggg                                           >  1:341616/1‑301 (MQ=255)`  
                                                                                                                                                                                                                                                                           `catGCCAGGGTATGACCGCCGTGTCAATGGTGGCATCGCCATCATGGGGTCTACTGGTAAAACCACTAAGGAACTTAAGGATGAGATTATGGCTCTAAAAGCGAAAGCTGAGGGAGACGGTAAGAAGACTGGCTAGGTACATGCTTTAATGGATACCGTTAAGGTTCTTACTGTTCGTGCTAGACGCAATCAGGACACTGTGTGGGAAACCTCACTGCGTGCCATCAATGACCTAGGGTTCTTCGCTAAGAACGCCTACATGGGTGCTCAGAACATTACGGAGATTGCCGGGATGATTGTc                                  <  1:100199/301‑1 (MQ=255)`  
                                                                                                                                                                                                                                                                            `atGCCAGCGTATGACCGCCGTGTCAATGGTGACATCGCCATCATGGGGTCTACTGGTAAAACCACTAAGGAACTTAATGATGAGATTTTGGCTCTCAAAGCGAAAGCTGAGGGAGACGGTAAGAAGACTGGCGAGGTACATGCTTTAATGGATACCGTTAAGATTCTTACTGGTCGTGCTAGACGCAATCAGGACACTGTGTGGGAAACCTCACTGCGTGCCATCAATGACCTAGGGTTCTTCGCTAAGAACGCCTACATGGGTGCTCAGAACATTACGGAGATTGCCGGGATGATTGTCa                                 >  1:198055/1‑301 (MQ=255)`  
                                                                                                                                                                                                                                                                            `atGCCAGCGTATGACCGCCGTGTCAATGGTGACATCGCCATCATGGGGTCTACTGGTAAAACCACTAAGGAACTTAAGGATGAGATTTTGGCTCTCAAAGCGAAAGCTGAGGGAGACGGTAAGAAGACTGGCGAGGTCCATGCTTTAATGGATACCGTTAAGATTCTTACTGGTCGTGCTCGACGCAATCAGGCCACTGTGTGGGAAACCTCACTGCGTTCCATCAATGACCTAGGGTTCTTCGCTAAGAACGCATACATGGGTGCTCAGAACATTACGGACATTGCTGGGGGGAATGTcc                                 >  1:327838/1‑300 (MQ=255)`  
                                                                                                                                                                                                                                                                                  `gCGTATGACCGCCGTGTCAATGGTGGCATCGCCATCATGGGGTCTACTGGTAAAACCACTAAGGAACTTAAGGATGAGATTTTGGCTCTCAAAGCGAAAGCTGAGGGAGACGGTAAGAAGACTGGCGAGGTACATGCTTTAATGGATACCGTTAAGATTCTTACTGGTCGTGCTAGACGCAATCAGGACACTGTGTGGGAAACCTCACTGCGTGCCATCAATGACCTAGGGTTCTTCGCTAAGAACGCCTACATGGGTGCTCAGAACATTACGGAGATTGCTGGGATGATTGTcctgtctc                           >  1:778190/1‑294 (MQ=255)`  
                                                                                                                                                                                                                                                                                   `agTATGACCGCCGTGTCAATGGTGACATCGCCATCATGGGGTCTACTGGTAAAACCACTAAGGAACTTAAGGATGAGATTTTGGCTCTCCAAGCGAAAGCTGAGGGAGACGGTAAGAAGACTGGCGAGGTACATGCTTTAATGGATACCGTTAAGATTCTTACTGGTCGTGCTAGACGCAATCAGGACACTGTGTGGGAAACCTCACTGCGTGCCATCAATGACCTAGGGTTCTTCGCTAAGAACGCCTACATGGGTGCTCAGAACATTACGGAGATTGCTGGGGTGATTGTCACTAGTaa                          <  1:527754/300‑1 (MQ=255)`  
                                                                                                                                                                                                                                                                                            `gccgTGTCAATGGTGACATCGCCATCATGGGGTCTACTGGTAAAACCACTAAGGAACTTAAGGATGAGATTTTGGCTCTCAAAGCGAAAGCTGAGGGAGACGGTAAGAAGACTGGCGAGGTACATGCTTTAATGGATACCGTTAAGATTCTTACTGGTCGTGCTAGACGCAATCAGGACACTGTGTGGGAAACCTCACTGCGTGCCATCAATGACCTAGGGTTCTTCGCTAAGAACGCCTACATGGGTGCTCAGAACATTACGGAGATTGCTGGGATGATTGTCACTGGTAACGTTCGTGc                 <  1:236225/301‑1 (MQ=255)`  
                                                                                                                                                                                                                                                                                                    `aaTGGTGGCATCGCCATCATGGGGTCTACTGGTAAAACCACTAAGGAACTTAAGGATGAGATTTTGGCTCTCAAAGCGAAAGCTGAGGGAGACGGTAAGAAGACTGGCGATGTACATGCTTTAATGGATACCGTTAAGATTCTTACTGGTCGTGCTAGACGCAATCAGGACACTGTGTGGGAAACCTCACTGCGTGCCATCAATGACCTAGGGTTCTTCGCTAAGAACGCCTACATGGGTGCTCAGAACATTACGGAGATTGCTGGGATGATTGTCACTGGTAACGTTCGTGCTCTAGGGc         <  1:520464/301‑1 (MQ=255)`  

                                                                                                                                                                                                                                                                                                           `|`                                                                                                                                                                                                                                                                                                              
`TGAGATGCTTAAGGAATTACACGGGGTGAAGGAAGTAACACCAGAGATGGTAGAGAAGTACGCTATGGATAAGGCTTATGGTATCTCCCACTCAGACCAGTTCACCAACAGTTCCATAATAGAAGAGAACATTGAGGGCTTAGTAGGTATCGAGAATAACTCATTCCTTGAGGCACGTAACTTGTTTGATTCGGACCTATCCATCACTATGCCAGACGGACAGCAATTCTCAGTGAATGACCTAAGGGACTTCGATATGTTCCGCATCATGCCAGCGTATGACCGCCGTGTCAATGGTGACATCGCCATCATGGGGTCTACTGGTAAAACCACTAAGGAACTTAAGGATGAGATTTTGGCTCTCAAAGCGAAAGCTGAGGGAGACGGTAAGAAGACTGGCGAGGTACATGCTTTAATGGATACCGTTAAGATTCTTACTGGTCGTGCTAGACGCAATCAGGACACTGTGTGGGAAACCTCACTGCGTGCCATCAATGACCTAGGGTTCTTCGCTAAGAACGCCTACATGGGTGCTCAGAACATTACGGAGATTGCTGGGATGATTGTCACTGGTAACGTTCGTGCTCTAGGGCATGGTAT  >  NC_001604.1/32724‑33323`

  

Alignment Legend

Aligned base mismatch/match (shaded by quality score): `ATCG/ATCG` `< 3 ≤` `ATCG/ATCG` `< 11 ≤` `ATCG/ATCG` `< 25 ≤` `ATCG/ATCG` `< 36 ≤` `ATCG/ATCG` `< 38 ≤` `ATCG/ATCG`

Unaligned base: `atcg`    Masked matching base: `atcg`    Alignment gap: `‑`    Deleted base: `‑`
